# Finding new medicines to fight CF: multiple steps of a success story

**DOI:** 10.1186/1750-1172-7-S2-A19

**Published:** 2012-11-22

**Authors:** Margarida D Amaral

**Affiliations:** 1 BioFIG-Center for Biodiversity, Functional and Integrative Genomics – Faculty of Sciences, University of Lisboa, Campo Grande, 1749-016 Lisboa, Portugal; 2 Centre of Human Genetics, National Institute of Health, Av. Padre Cruz, 1649-016 Lisboa, Portugal

## Background

Cystic fibrosis (CF) is a major life-limiting genetic disease leading to severe respiratory symptoms caused by mutations in CF trans membrane conductance regulator (CFTR), a chloride channel expressed at the apical membrane of epithelial cells. Absence of functional CFTR from the surface of respiratory cells reduces mucociliary clearance, promoting airways obstruction, chronic infection and ultimately lung failure [1]. Despite major clinical advances treating the symptoms, which pushed survival beyond the second decade (~25 years in Europe), CF is still a life-limiting condition [2]. However, to further increase CF patients life expectancy, CF needs to be treated beyond its symptoms, i.e., through treatments addressing the basic defect associated with CFTR gene mutations [3]. So far ~1,900 CFTR mutations were reported [4], but one single mutation, F508del remains the most common one, as it occurs in ~90% of CF patients in at least one allele [5] and is associated with a severe clinical phenotype. Despite that most of efforts are focused on correcting the F508del-CFTR which causes intracellular retention of the mutant channel at the endoplasmic reticulum (ER), several additional strategies are emerging to rescue other (rarer mutants) which, in some populations, also have high prevalence. To this end, CFTR mutations are usually grouped into functional classes, towards a "mutation-specific" therapeutic approach by which mutations within the same functional class can be corrected by the same therapeutic strategy towards a "personalized medicine" approach [6].

## Materials and methods/ results

To apply such strategy CFTR mutations are thus classified into six main functional categories [7,8], namely: *i*) class I mutations (often mutations generating premature stop codons, e.g., R1162X) prevent protein production; *ii*) class II mutations (includes F508del) cause intracellular retention and premature degradation, thus preventing mutant CFTR from reaching the cell surface; *iii*) class III mutants (e.g., G551D) cause impairment in the channel gating (i.e., decreased open probability); *iv*) class IV mutants have substantially reduced flow of Cl^-^ ions through the CFTR channel (e.g., R334W); *v*) class V mutants include mostly alternative splicing mutants (e.g., 3272-26A>G) which allow synthesis of some normal CFTR mRNA (and protein), albeit at very low levels; and *vi*) class VI mutants (e.g., c.120del23 [9] or membrane-rescued F508del) impair the plasma membrane stability of CFTR.

## Conclusions

Several therapeutic strategies adopting this "mutation-specific" approach are currently under experimental testing or clinical trial [3,6]. Based on the current "drug pipeline", these are expected to rise in numbers very soon.
